# Delineation of a *KDM2B*-related neurodevelopmental disorder and its associated DNA methylation signature

**DOI:** 10.1016/j.gim.2022.09.006

**Published:** 2022-11-01

**Authors:** Richard H. van Jaarsveld, Jack Reilly, Marie-Claire Cornips, Michael A. Hadders, Emanuele Agolini, Priyanka Ahimaz, Kwame Anyane-Yeboa, Severine Audebert Bellanger, Ellen van Binsbergen, Marie-Jose van den Boogaard, Elise Brischoux-Boucher, Raymond C. Caylor, Andrea Ciolfi, Ton A.J. van Essen, Paolo Fontana, Saskia Hopman, Maria Iascone, Margaret M. Javier, Erik-Jan Kamsteeg, Jennifer Kerkhof, Jun Kido, Hyung-Goo Kim, Tjitske Kleefstra, Fortunato Lonardo, Abbe Lai, Dorit Lev, Michael A. Levy, M.E. Suzanne Lewis, Angie Lichty, Marcel M.A.M. Mannens, Naomichi Matsumoto, Idit Maya, Haley McConkey, Andre Megarbane, Vincent Michaud, Evelina Miele, Marcello Niceta, Antonio Novelli, Roberta Onesimo, Rolph Pfundt, Bernt Popp, Eloise Prijoles, Raissa Relator, Sylvia Redon, Dmitrijs Rots, Karen Rouault, Ken Saida, Jolanda Schieving, Marco Tartaglia, Romano Tenconi, Kevin Uguen, Nienke Verbeek, Christopher A. Walsh, Keren Yosovich, Christopher J. Yuskaitis, Giuseppe Zampino, Bekim Sadikovic, Mariëlle Alders, Renske Oegema

**Affiliations:** 1 Department of Genetics, University Medical Center Utrecht, Utrecht, the Netherlands;; 2 Department of Pathology and Laboratory Medicine, Western University, London, Ontario, Canada;; 3 Oncode Institute and Center for Molecular Medicine, University Medical Center Utrecht, Utrecht University, Utrecht, The Netherlands;; 4 Laboratory of Medical Genetics, Translational Cytogenomics Research Unit, Bambino Gesù Children Hospital, IRCCS, 00165 Rome, Italy;; 5 Division of Clinical Genetics, Department of Pediatrics, Columbia University, New York, NY;; 6 Service de Génétique Médicale et de Biologie de la Reproduction, Centre Hospitalier Regional Universitaire Brest, Brest, France;; 7 Centre de Génétique Humaine, CHU de Besançon, Université de Franche-Comté, Besançon, France;; 8 Greenwood Genetic Center, Greenwood, SC;; 9 Genetics and Rare Diseases Research Division, Bambino Gesù Children’s Hospital, IRCCS, Rome, Italy;; 10 Department of Medical Genetics, University Medical Centre Groningen, University of Groningen, Groningen, The Netherlands;; 11 Medical Genetics Unit, A.O.R.N. San Pio, Benevento, Italy;; 12 Laboratorio di Genetica Medica - ASST Papa Giovanni XXIII, Bergamo, Italy;; 13 Department of Medical Genetics, BC Children’s Hospital Research Institute, The University of British Columbia, Vancouver, British Columbia, Canada;; 14 Department of Human Genetics, Radboud University Medical Center, Nijmegen, the Netherlands;; 15 Verspeeten Clinical Genome Centre, London Health Sciences Centre, London, Ontario, Canada;; 16 Department of Pediatrics, Faculty of Life Sciences, Kumamoto University, Kumamoto, Japan;; 17 Neurological Disorders Research Center, Qatar Biomedical Research Institute, Hamad Bin Khalifa University, Doha, Qatar;; 18 Division of Epilepsy and Clinical Neurophysiology and Epilepsy Genetics Program and Genetics and Genomics, Boston Children’s Hospital, Harvard Medical School, Boston, MA;; 19 The Rina Mor Institute of Medical Genetics, Wolfson Medical Center, Holon, Israel;; 20 Division of Genetics and Genomics and Howard Hughes Medical Institute, Boston Children’s Hospital, Boston, MA;; 21 Department of Human Genetics, Yokohama City University Graduate School of Medicine, Yokohama, Japan;; 22 The Raphael Recanati Genetic Institute, Rabin Medical Center, Beilinson Hospital, Petach-Tikva, Israel;; 23 Sackler Faculty of Medicine, Tel Aviv University, Tel Aviv, Israel;; 24 Department of Human Genetics, Gilbert and Rose-Marie Chagoury School of Medicine, Lebanese American University, Beirut, Lebanon;; 25 Institut Jérôme Lejeune, Paris, France;; 26 Department of Medical Genetics, CHU Bordeaux, Bordeaux, France;; 27 Department of Pediatric Hematology and Oncology and Cellular and Gene Therapy, Bambino Gesù Children’s Hospital, Scientific Institute for Research, Hospitalization and Healthcare (IRCCS), Rome, Italy;; 28 Center for Rare Diseases and Congenital Defects, Fondazione Policlinico Universitario A. Gemelli IRCCS, Rome, Italy;; 29 Institute of Human Genetics, University of Leipzig Medical Center, Leipzig, Germany;; 30 Center of Functional Genomics, Berlin Institute of Health at Charité – Universitätsmedizin Berlin, Berlin, Germany;; 31 Université de Brest, Inserm, EFS, UMR 1078, GGB, Brest, France;; 32 Department of Pediatric Neurology, Radboud University Medical Center, Nijmegen, the Netherlands;; 33 Clinical Genetics Unit, Department of Women and Children’s Health, University of Padova, Padova, Italy;; 34 Molecular Genetic Laboratory, Edith Wolfson Medical Center, Holon, Israel;; 35 Faculty of Medicine and Surgery, Catholic University of Sacred Heart, Rome, Italy;; 36 Department of Human Genetics, Amsterdam UMC, University of Amsterdam, Amsterdam Reproduction and Development Research Institute, Amsterdam, The Netherlands;; 37 Division of Epilepsy and Clinical Neurophysiology and Epilepsy Genetics Program, Boston Children’s Hospital, Harvard Medical School, Boston, MA;

**Keywords:** Human Genetics, KDM2B, MDEMs, Methylation signatures, Neurodevelopmental disorders

## Abstract

**Purpose:**

Pathogenic variants in genes involved in the epigenetic machinery are an emerging cause of neurodevelopment disorders (NDDs). Lysine-demethylase 2B (*KDM2B*) encodes an epigenetic regulator and mouse models suggest an important role during development. We set out to determine whether *KDM2B* variants are associated with NDD.

**Methods:**

Through international collaborations, we collected data on individuals with heterozygous *KDM2B* variants. We applied methylation arrays on peripheral blood DNA samples to determine a *KDM2B* associated epigenetic signature.

**Results:**

We recruited a total of 27 individuals with heterozygous variants in *KDM2B*. We present evidence, including a shared epigenetic signature, to support a pathogenic classification of 15 *KDM2B* variants and identify the CxxC domain as a mutational hotspot. Both loss-of-function and CxxC-domain missense variants present with a specific subepisignature. Moreover, the *KDM2B* episignature was identified in the context of a dual molecular diagnosis in multiple individuals. Our efforts resulted in a cohort of 21 individuals with heterozygous (likely) pathogenic variants. Individuals in this cohort present with developmental delay and/or intellectual disability; autism; attention deficit disorder/attention deficit hyperactivity disorder; congenital organ anomalies mainly of the heart, eyes, and urogenital system; and subtle facial dysmorphism.

**Conclusion:**

Pathogenic heterozygous variants in *KDM2B* are associated with NDD and a specific epigenetic signature detectable in peripheral blood.

## Introduction

Genes encoding for epigenetic regulators are an emerging class of monogenic disease genes associated with neurodevelopment disorders (NDDs). This group of disorders, collectively referred to as “Mendelian Disorders of the Epigenetic Machinery” (MDEMs), present with neurodevelopmental delay, congenital malformations, and/or growth abnormalities.^1^ For an increasing number of MDEMs, distinct genome-wide methylation signatures (episignatures) have been identified.^2^ These signatures present as valuable tools in clinical practice because they are unique for each disorder and can be detected in peripheral blood samples, providing a robust and easily accessible diagnostic tool that enables the diagnosis of uncharacterized individuals as well as the identification of novel pathogenic variants through pinpointing the causal gene.^3^

The *KDM2B* gene (lysine-demethylase 2B, also called FBXL10, NDY1, CXXC2, and JHDM1B; OMIM 609078) encodes for a well-studied component of the epigenetic machinery. The canonical, full-length KDM2B protein acts by demethylating lysine residues K4, K36, and K79 of histone 3.^4–11^ This catalytic activity is provided by the JmjC domain, which is conserved from yeast to human.^4,9^ The CxxC domain directs KDM2B to promotor regions by binding unmethylated CpG dinucleotides.^12–14^ This DNA-binding capacity has been linked to the recruitment of the polycomb repressive complex 1 to developmental genes.^10,15,16^ *KDM2B* has been implicated in many biological processes, including cell cycle regulation, metabolic regulation, and DNA-damage repair.^11,13,17,18^ Moreover, in line with a central role in epigenetic and transcriptional regulation, *KDM2B* is essential for organism development and regulates cellular differentiation.^12^

*KDM2B*, together with *SETD1B*, has been implicated as a disease-causing gene in the rare 12q24.31 microdeletion syndrome.^19–21^ In addition, 2 sporadic patients and 1 family with heterozygous *KDM2B* missense variants have been described.^22–24^ A monogenic *KDM2B*-related human disorder has however not been delineated, and the significance of the reported variants remains uncertain.

## Materials and Methods

### Inclusion criteria and data collection

Individuals were included based on the identification of a heterozygous *KDM2B* suspected to be pathogenic on the basis of in silico predictions and/or inheritance. Individuals carrying biallelic variants of uncertain significance (VUS) were not considered for this study. Individual 1 was identified as the index patient after a *KDM2B* variant was annotated to be of interest after diagnostic trio exome sequencing. Families 2 to 5 were included after local, in-house database searches. All remaining individuals were included after personal communication, literature search, or from searches using the GeneMatcher platform.^25^ For the published cases, we contacted the original authors for updated clinical information. For all individuals, clinical and genetic data were collected through a standardized spreadsheet, which was completed by the respective physicians and/or researchers.

### Genetic variant detection

Variants in individuals/families 24, 25, 29, 30, and 34 were identified as described before.^19,20,22,24^ The variant in individual 4.3 was identified through targeted Sanger sequencing. All other variants were detected through clinical and/or research-based exome sequencing.

### Analysis and classification of *KD2MB* variants

Structural analysis of variants was performed using Pymol (The Pymol Molecular Graphics System, Version 2.5, Schrödinger, LLC). All Figures were generated using Pymol. Four in silico prediction algorithms were consulted: Sorting Intolerant from Tolerant, Metadome, MutationTaster, and Polymorphism Phenotyping v2.^26–29^ All variants were manually analyzed using Alamut Visual v2.15 (Sophia Genetics). Variants were classified according to the 2015 American College of Medical Genetics and Genomics/Association for Molecular Pathology guidelines.^30^ Episign results were used to support classification according to criterium Pathogenic Strong 3 (PS3), and Pathogenic Moderate 1 (PM1) was applied for variants in the CxxC domain.

### Development of episignature

Materials and methods associated with episignature development are provided as Supplemental Methods in the supplementary material.

## Results

**Figure 1:**
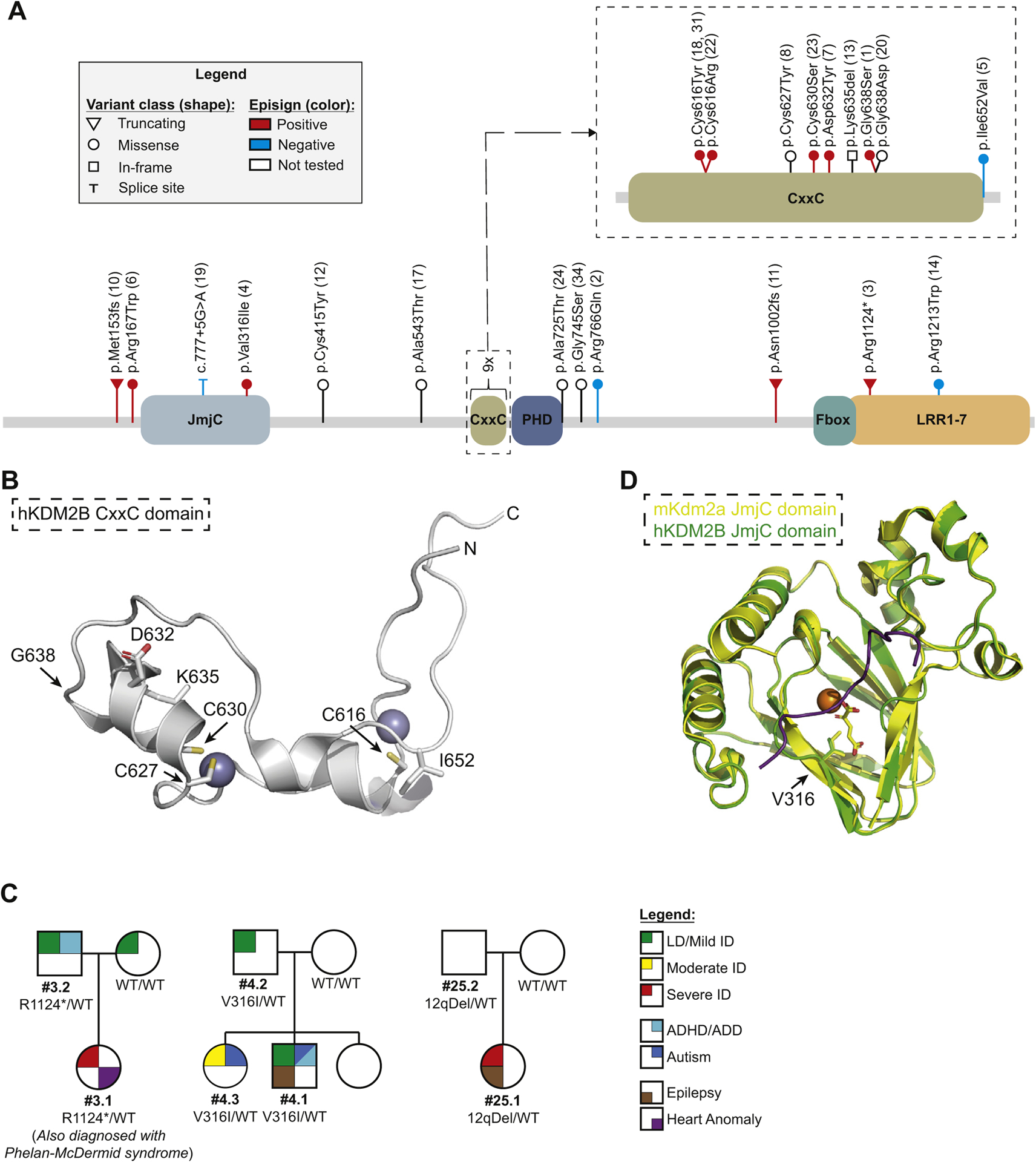
A cohort of individuals with heterozygous *KDM2B* variants. A. Schematic representation of the *KDM2B* gene, its known domains, and the variants included in this study. Lollipops representing individual variants indicate location, classification of (predicted) effect on the transcript and/or protein (shape), and the classification based on the first analysis of the methylation arrays (color; Supplemental Figure 2). Larger deletions (ie, cases 25.1, 25.2, 29, and 30) are not shown. B. Projection of CxxC domain missense variants on the known crystal structure. Purple spheres represent Zn^2+^ ions. Side chains of relevant residues are included. C. Pedigrees depicting all cases of inherited pathogenic variants for which the pedigrees have not been published before (families 3, 4, and 25). All remaining pedigrees can be found in Supplemental Figure 1. D. Projection of the p.Val316Ile (family 4) variant on the structure of the mouse Kdm2a JmjC structure (yellow). Predicted human KDM2B JmjC structure as determined by AlphaFold is shown in green. Orange sphere indicates Fe^2+^ ion and the α-ketoglutarate cofactor is shown as yellow sticks. Purple line indicates target peptide (histon 3). ADD, attention deficit disorder; ADHD, attention deficit hyperactivity disorder; ID, intellectual disability; LD, learning difficulties; WT, wild type.

We initiated this study after the identification of a de novo c.1912G>A (p.Gly638Ser) variant in *KDM2B* (NM_032590.4; Table 1; Supplemental Table 1) through diagnostic trio exome sequencing in the index patient (individual 1), who was diagnosed with speech delay, autism spectrum disorder (ASD), and a congenital heart defect (CHD) (Table 2; Supplemental Table 2). The identified variant was absent from the Genome Aggregation Database (gnomAD),^31^ predicted damaging by multiple algorithms (Supplemental Table 1), and affects a well-conserved residue (Supplemental Figure 1A) located within the CxxC domain (Figure 1A and B). *KDM2B* presented as an outstanding candidate disease gene because the gene is intolerant for both putative loss-of-function (LoF) (observed/expected = 0.09 [0.05–0.18]) and missense (*z*-score = 3.44) variants in the general population.^31^ In addition, several animal models presented with severe congenital defects.^12,32–35^ We therefore aimed to study additional individuals with heterozygous *KDM2B* variants and formed this cohort after online matchmaking using the GeneMatcher platform,^25^ literature search, personal communication, and in-house database searches. This study was approved by the medical ethical committee installed by the University Medical Centre Utrecht (TCBIO 20/714, March 18, 2021).

We collected data of a total of 27 individuals from 22 families representing 21 different heterozygous variants in *KDM2B* (Figure 1; Tables 1 and 2; Supplemental Figure 1; Supplemental Tables 1 and 2). Our cohort encompassed 3 putative LoF variants, 13 missense variants, 1 in-frame deletion, and three 12q24.31 microdeletions. A total of 17 variants were confirmed to be de novo. Of note, one of these variants was identified twice and thus occurred de novo at 2 independent occasions (c.1847G>A; individuals 18 and 31). Four variants were inherited (families 3, 4, 5, and 25; Figure 1C and Supplemental Figure 1B). Four individuals with microdeletions have been reported previously: family 25 with an inherited 12q deletion^19^ and individuals 29 and 30 with a de novo 12q deletion including *KDM2B* and *SETD1B*.^20^

We observed a remarkable clustering of variants (8 missense and 1 in-frame deletion) in the DNA-binding CxxC domain (Figure 1A and B), of which, 7 missense variants were predicted damaging by all algorithms. The only exception was p.Ile652Val, which was the only inherited variant and is reported twice in gnomAD. We performed structural modeling, which showed that this residue is located toward the surface and the substitution is not expected to influence local structure. All other variants affecting the CxxC domain however are expected to affect protein function by either interfering with the binding with Zn^2+^ ions (p.Cys616, p.Cys627, and p.Cys630) or affecting the local structure (p.Gly638, p.Asp632, and p.Lys635; Figure 1B). The p.Val316Ile variant is likely located at the active site of the JmjC domain and therefore is expected to affect its catalytic function (Figure 1D).

Because of the role of KDM2B in the epigenetic machinery, we hypothesized that impaired KDM2B function leads to genome-wide changes in DNA methylation, an effect that has been observed in >50 other genetic syndromes.^2,3,36^ These methylation changes present as disease specific episignatures, which are detectable in peripheral blood. As such, episignatures provide not only fundamental insights into the molecular consequences of genetic variants but also easily accessible diagnostic tools to identify syndromes or reclassify VUS.^3^

We generated genome-wide methylation array data for 21 individuals (Table 1; Supplemental Table 1) according to the previously established protocols.^37^ We excluded 2 samples because of technical errors (samples 2 and 4.2; Supplemental Figure 2D). Another 3 samples failed to group with case samples after cross validations and were excluded for the establishment of the episignature (samples 5.1, 14, and 19; Supplemental Figure 2B, D, and E). These 3 variants were previously classified as VUS on the basis of their inheritance and/or presence in gnomAD (Table 1; Supplemental Table 1).

**Figure 2:**
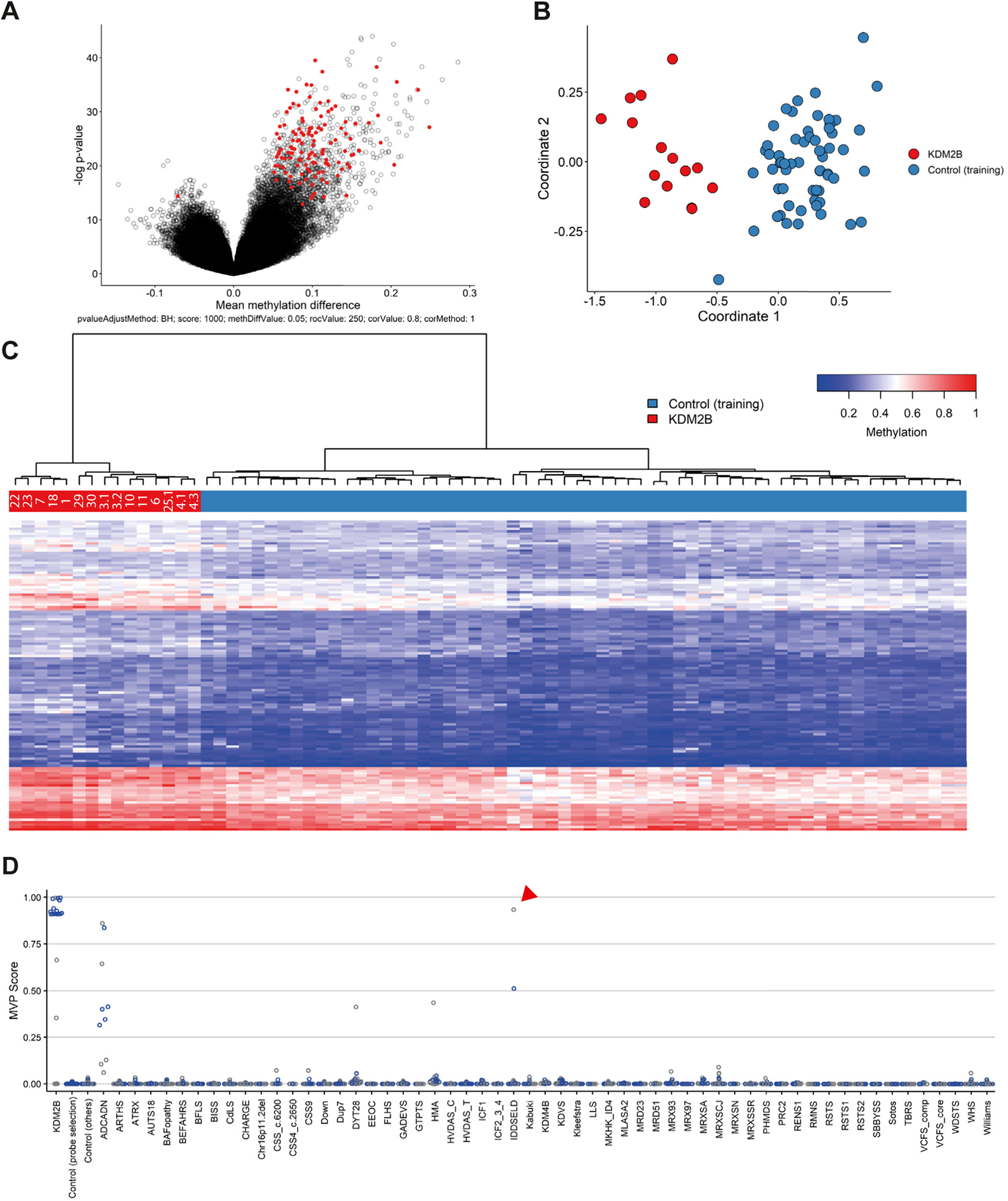
A *KDM2B* specific episignature. After initial analysis (Supplemental Figure 2), 15 samples identified as outliers in the initial analysis were included for the training of a *KDM2B* specific episignature. A. Volcano plot indicating probes (red) included in the *KDM2B* episignature. B. Multidimensional scaling plot for selected probes, representing the pairwise distance across samples (red) and controls (blue) based on the top 2 dimensions. C. Heatmap of selected probes and unsupervised hierarchal clustering results indicating the episignature’s ability to differentiate *KDM2B* variants (red) from controls (blue). D. Support vector machine (SVM) classifier indicating specificity of the *KDM2B* episignature. Graph shows summary of 4-fold validation using all 15 case samples, using 75% of unaffected controls and other episignatures for training (blue) and the other 25% for testing (gray). Y-axis: MVP scores as determined by SVM. X-axis: different groups of samples, controls, and other known episignatures. Red arrowhead indicates the intellectual developmental disorder with seizures and language delay sample referred to in the text. MVP, methylation variant pathogenicity.

The remaining 15 samples, representing 13 variants, were used to establish a *KDM2B* episignature (Figure 2). Methylation patterns were assessed for sample quality, degree of methylation change, and statistical robustness of observed changes at each probe, allowing for effective modeling of the methylation differences observed between case samples and matched controls (see Materials and Methods). Comparisons were performed against age- and sex-matched controls, leading to the identification of 156 statistically differentially methylated probes (Figure 2A). Hierarchical clustering based on this probe set showed distinct clustering of case samples away from controls, with all samples presenting a more similar methylation profile to one another than the matched controls (Figure 2B and C). Cross validation assays, based on the removal of each single sample from the probe selection training process, confirmed that the probe set was able to effectively identify *KDM2B* variants because all case samples remained grouped together in each iteration (Supplemental Figure 3B). In conclusion, we have established an episignature that is able to discriminate *KDM2B* variants from controls and classified these 13 variants as pathogenic (Table 1). Interestingly, the *KDM2B* associated episignature mainly consisted of hypermethylated probes (Figure 2A).

We next tested the sensitivity and specificity of the episignature using a support vector machine. For each sample, we determined a methylation variant pathogenicity (MVP) score between 0 and 1 on the basis of matching the *KDM2B* episignature. All *KDM2B* samples included in the training set received scores of >0.8, whereas control samples remained near 0, indicating high sensitivity for the detection of the *KDM2B* episignature (Supplemental Figure 3C). Specificity was tested using a similar classifier that was instead trained against a large number of samples with confirmed diagnoses of non-*KDM2B* related disorders from our Episign knowledge database. In total, 75% of both case and control samples were used for training the classifier with the remaining 25% reserved for testing (Figure 2D). Case samples again scored high (>0.85) whereas the remainder of samples scored low (<0.5), with few exceptions. The most notable exceptions were cerebellar ataxia, deafness, and narcolepsy (ADCADN; OMIM 604121), Hunter-McAlpine syndrome (HMA; OMIM 601379), and dystonia 28, childhood-onset (DYT28; OMIM 617284). One other sample among the control samples did score remarkably high for the *KDM2B* signature (Figure 2D, red arrow head). This patient was previously diagnosed with intellectual developmental disorder with seizures and language delay (IDDSELD; OMIM 619000), a disorder caused by *SETD1B* variants. Upon closer investigation, we identified this sample to originate from a case with a 12q24.31 microdeletion including *SETD1B* but not *KDM2B*.^20,38^ We hypothesize that the deletion might have affected *KDM2B* regulatory regions. For reference, we have included the clinical description of this individual (individual 33, Supplemental Table 2).

A total of 4 *KDM2B* variants within our cohort tested negative for the signature. Among the negative samples are 3 missense variants for which pathogenicity was doubtful based on the a priori predictions and/or gnomAD data. The other negative sample in our cohort was that of the only splice-site variant, indicating that the predicted splice effects do not occur or at least not to a level that interferes with gene functionality. We classified these 4 variants as VUS because a negative episignature result does not suffice to infer an absence of functional effects (Table 1).

Of note, 2 individuals in our cohort with microdeletions encompassing both *KDM2B* and *SETD1B* (individuals 29 and 30) were previously shown to have the *SETD1B*-associated episignature.^20^ In the same samples, we have now additionally identified the *KDM2B* episignature. Another sample showing 2 distinct episignatures was sample 3.1, which was from a girl who was previously diagnosed with Phelan-McDermid syndrome (OMIM 606232) due to a 22q13 deletion. These results indicate that multiple episignatures can coexist in a single individual, and the method is able to correctly identify 2 syndromes independently.

**Figure 3:**
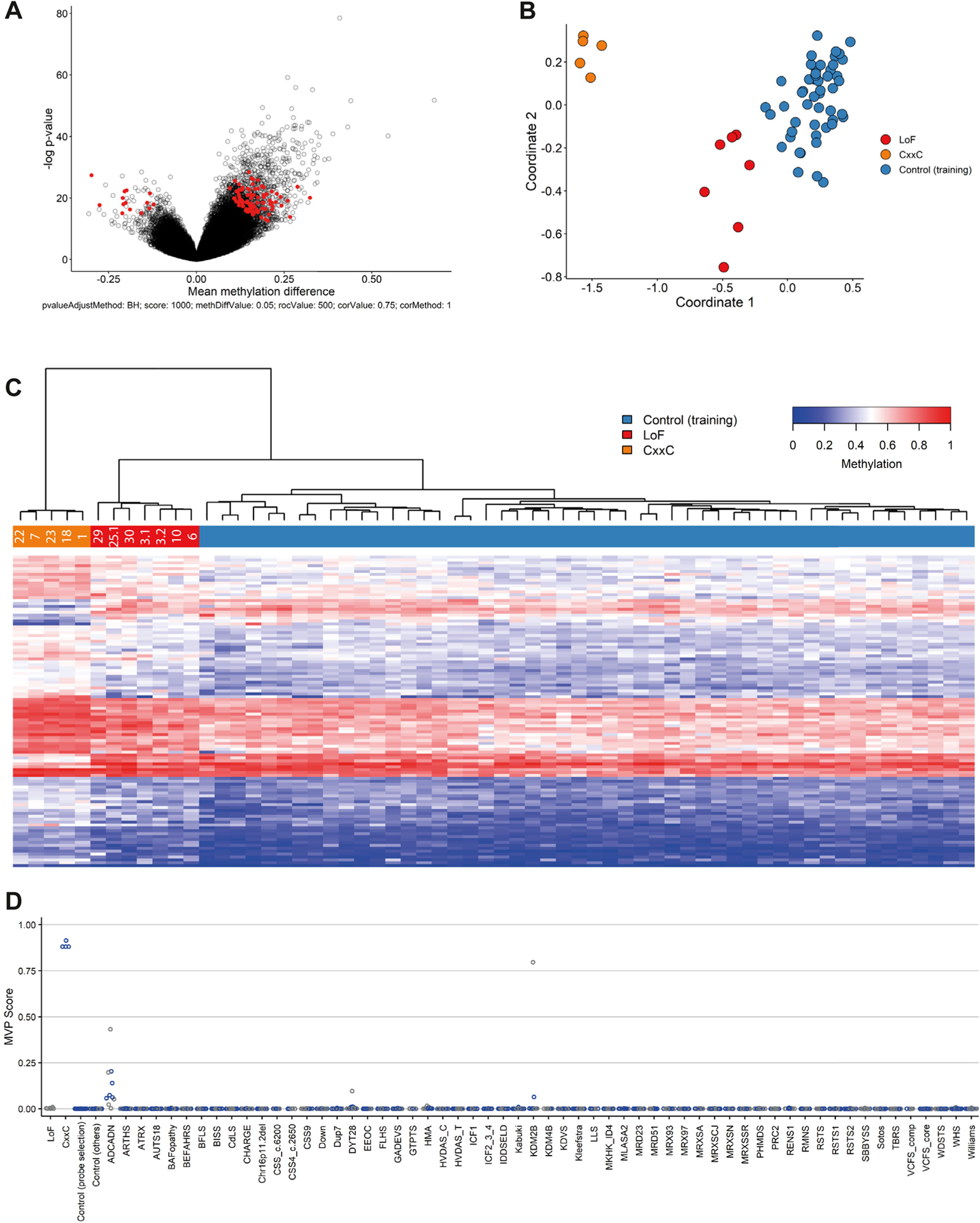
A CxxC-variant specific episignature. All samples representing a CxxC-domain variant and included in the *KDM2B* episignature training set were used to train a CxxC-variant specific episignature. A. Volcano plot indicating all probes (red) included in the CxxC episignature. B. Multidimensional scaling plot for selected probes representing the pairwise distance across CxxC variants (orange), LoF variants (red), and controls (blue) based on the top 2 dimensions. C. Heatmap of selected probes and unsupervised hierarchal clustering results indicating the episignature’s ability to differentiate *KDM2B* variants (red and orange) from controls (blue) and to differentiate CxxC variants (orange) from LoF variants (red). D. Support vector machine classifier indicating specificity of the CxxC episignature. Graph is same as in Figure 2D. MVP, methylation variant pathogenicity.

The clustering of missense variants in the CxxC domain suggests a distinct effect on KDM2B function over LoF variants. We therefore trained a separate classifier using 5 CxxC domain missense samples (Figure 3; Supplemental Table 1). Interestingly, the resulting probeset not only correctly differentiated all *KDM2B* variants (ie, including the LoF variants) from controls but also was able to distinguish CxxC and LoF variants (Figure 3B and C). Of note, 106 hypermethylated probes among the 107 significant probes selected for the CxxC-trained episignature present with an on average increased methylation level, even exceeding that of the hypermethylated probes of the pan-*KDM2B* probeset (mean methylation difference of all hypermethylated probes: 16.56% ± 4.21% vs 10.38% ± 3.68%; Figure 2A and Figure 3A). Importantly, a probeset trained using LoF samples was able to discriminate CxxC from LoF variants as well (Supplemental Figure 4). In conclusion, CxxC missense variants cause a distinct episignature that is associated with increased hypermethylation levels, suggesting an additional effect on KDM2B functioning of these variants.

Next, we set out to reclassify 4 variants that were not tested for the episignature on the basis of the American College of Medical Genetics and Genomics/Association for Molecular Pathology guidelines^30^ (Supplemental Table 1). Importantly, we considered the CxxC domain as an established hotspot for pathogenic variation in *KDM2B* (criterium PM1). We reclassified 2 variants as pathogenic, 1 variant as likely pathogenic, and 1 as VUS (Table 1; Supplemental Table 1). In summary, we classified 15 variants as pathogenic, 1 variant as likely pathogenic, and 6 variants remained as VUS (Table 1; Supplemental Table 1, Supplemental Figure 5).

**Figure 4:**
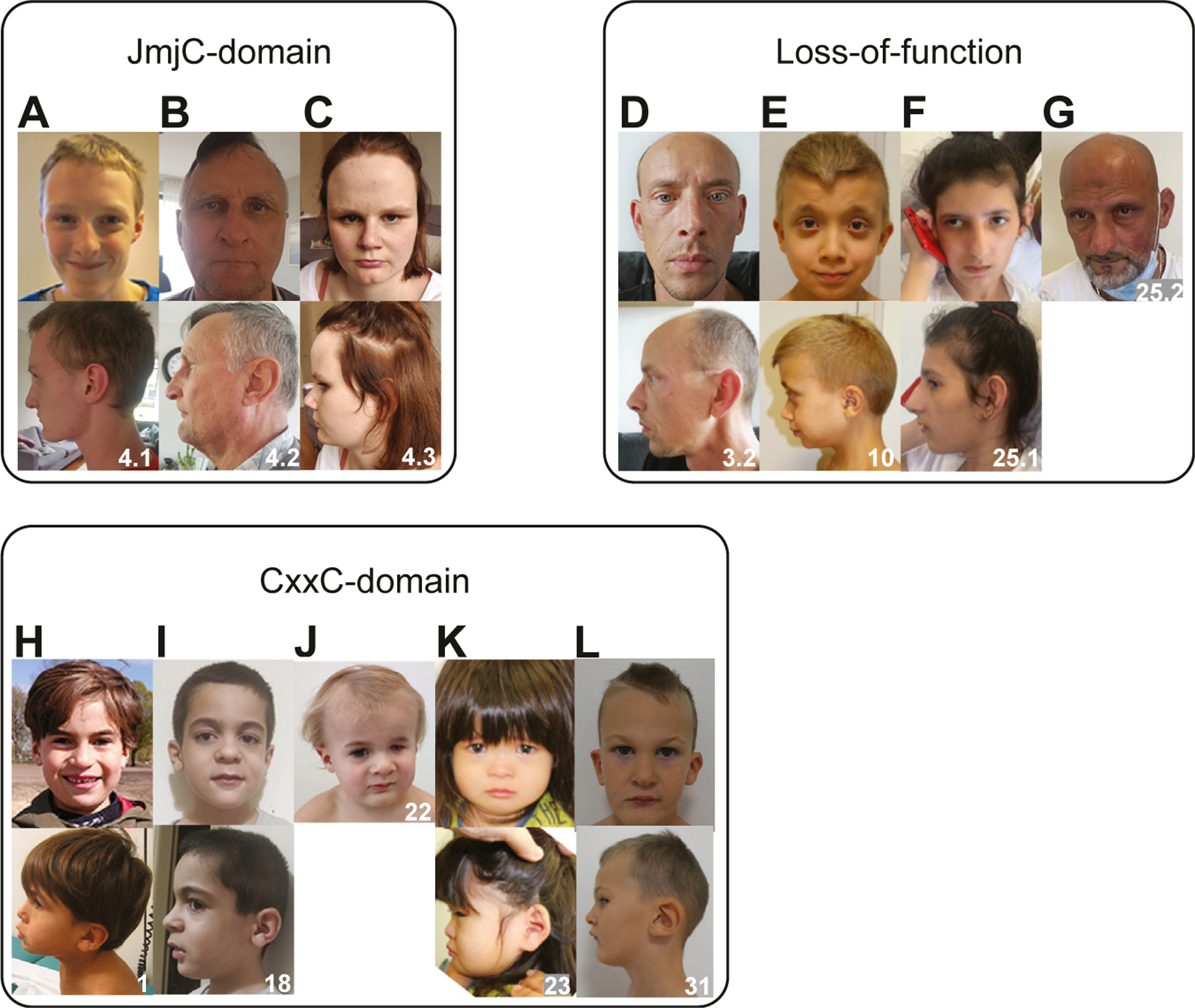
Facial characteristics of individuals with *KDM2B* pathogenic variants. A-C. Individuals of family 4 with the p.Val316Ile variant located in the JmjC-domain. D-G. Individuals with loss-of-function variants. Individual 10 (E) was also affected with Noonan syndrome. H-L. Individuals with missense variants in the CxxC domain.

Clinical data of all individuals are systematically presented in Table 2. A more detailed description for each individual and their clinical histories are provided as supplemental material. Data form previously described individuals are included in Supplemental Table 2 as well. Of the 21 individuals with a—likely—pathogenic variant, 4 had a microdeletion and 2 had an additional genetic diagnosis. The remaining 15 individuals all presented with speech delay, developmental delay (DD), learning difficulties, and/or intellectual disability (ID). Behavioral concerns such as ASD and attention deficit hyperactivity disorder were common (9/15). Growth parameters were within the normal range for most patients. We observe several congenital defects, including heart defects (7/15), unilateral kidney agenesis (4/15), and ophthalmological anomalies (6/14). Two patients had cryptorchidism and 2 had epilepsy. We collected facial photographs of 12 (12/21) individuals (Figure 4). Facial features noted in several individuals with CxxC-domain variants were a broad nasal tip, large ear lobes, and exaggerated Cupid’s bow. Interestingly, in the individuals with LoF variants the nose was often more prominent, with a narrow nasal ridge and malar flattening, with the exception of individual 10 who also had a diagnosis of Noonan syndrome. Despite these subtle features, no consistent facial gestalt could be identified. In summary, we have delineated a novel syndrome that is caused by heterozygous *KDM2B* variants and characterized clinically by DD/ID; behavioral challenges including autism and attention deficit hyperactivity disorder; congenital anomalies mainly of the heart, urogenital system, and eyes; and variable facial dysmorphism.

Because our methylation analysis revealed differences between CxxC and LoF variants, we studied potential genotype–phenotype relationships for these subgroups. Unilateral kidney agenesis and eye anomalies were only reported in CxxC cases. In addition, CHDs were present in 6 individuals with a CxxC variant (6/9) and only in 2 individuals with a LoF variant (2/8). Of note, this involved 1 individual with Noonan syndrome and 1 with Phelan-McDermid syndrome, and both syndromes are associated with CHD. We thus note that congenital organ anomalies might be overrepresented in individuals with CxxC variants; however, the current limited number of available cases precludes to draw any conclusions. Epilepsy did not occur in association with the CxxC-domain variants but occurred in 1 patient with a JmjC-domain variant, 1 patient with a frame-shift variant, and 2 patients with a 12q24.31 microdeletion.

## Discussion

We describe a novel NDD caused by heterozygous pathogenic variants in *KDM2B* and present an episignature associated with the disorder. We identify the CxxC domain as a mutational hotspot, and variants affecting this domain are associated with a specific episignature. In line with other MDEMs, individuals with pathogenic variants in *KDM2B* present with variable phenotypic expression, including DD/ID, congenital organ anomalies, and/or facial dysmorphisms. Most pathogenic variants are of de novo origin, however, in 3 families, the variant was found to be inherited. Interestingly, individual 25.2 is an adult male with a 12q24.31 microdeletion, who did not seem to be clinically affected even though the *KDM2B* episignature was present in his sample (data not shown). These observations might reflect the variable expression of this disorder; although, we also consider alternative hypotheses. Because we were unable to test the grandparents, we cannot exclude germline mosaicism. Alternatively, all inherited variants originated from the father, possibly indicating that males are less severely affected. In mice, *Kdm2b* has been shown to be involved in X-chromosome silencing,^39^ and as such, a different clinical expression in males vs females seems plausible. Larger cohorts are needed to fully encompass the phenotypes and penetrance associated with the disorder and refine its (domain) specific episignature(s).

For the *KDM2B* episignature, we noticed elevated MVP scores for 3 other disorders (Figure 2D). The first was ADCADN, caused by pathogenic variants in *DNMT1*, a methyltransferase known as the central player in the maintenance of CpG methylation.^2,40^ Interestingly, *DNMT1* has been suggested to regulate H3K4 methylation, providing a direct mechanistic link with *KDM2B*.^41^ The second was HMA, a syndrome associated with duplication of 5q35.^42,43^ This region includes *NSD1*, which encodes a lysine methyl transferase known to methylate H3K36,^44^ providing a direct functional link with *KDM2B* as well. Finally, DYT28 is associated with *KMT2B*,^45^ encoding another methyltransferase reported to methylate H3K4.^46^ Phenotypically, the *KDM2B* related disorder shares features with HMA, eg, mild to moderate developmental delay, CHDs, and dysmorphism, but presents differently from ADCADN and DYT28. Interestingly, the *KDM2B* related disorder, ADCADN, HMA, and DYT28 episignatures are all characterized by hypermethylation.^2,47,48^ The elevated MVP scores might therefore reflect a set of loci sensitive to hypermethylation, irrespective of the underlying mechanisms. Future studies will have to determine whether and how the *KDM2B* disorder, ADCADN, DYT28, and HMA share a common etiology.

Hypermethylation has previously been linked to *KDM2B* because loss of *Kdm2b* in mouse embryonic stem cells leads to genome-wide hypermethylation of promotors associated with polycomb repressive complexes, implying that Kdm2b protects against de novo DNA methylation during mouse development.^12^ Interestingly, re-expression of a short Kdm2b isoform, lacking the catalytic JmjC domain, resets the methylation of CpG-islands to baseline levels and rescues embryonic lethality.^12^ This short isoform is highly expressed in mouse embryonic stem cells,^34^ suggesting important functions of *Kdm2b* besides lysine demethylation activity.

The clustering of variants in the CxxC domain, an excess of congenital defects, and a distinct episignature with even further elevated levels of hypermethylation associated with CxxC-domain variants are suggestive of a—partial—dominant-negative effect of these variants. Interestingly, the CxxC domain has been specifically implicated in the developmental functions of *KDM2B* because CxxC-domain mutants fail to rescue cellular differentiation induced by *Kdm2b* depletion in mouse embryonic stem cells,^10^ and specific deletion of the CxxC domain induces developmental defects in the heterozygous state whereas heterozygous knock-outs appear healthy.^32,33^ Recently, a heterozygous conditional mutant mouse with loss of the CxxC domain in the developing brain was shown to exhibit impaired memory and ASD-like behaviors.^49^ These animal models therefore share many features with the human syndrome and also point to the CxxC domain harboring important developmental functions. Further studies are necessary to fully understand the broad effect of *KDM2B* on human development.

The *KDM2B*-associated NDD represents a novel addition to the emerging group of MDEMs.^1^ Its associated episignature can aid in reclassification of VUS and the detection of missed variants during routine diagnostic testing, eg, due to variants outside coding regions or due to inherited variants missed by standard trio filtering.

## Supplementary Material

Supplementary Material

Supplemental Table 2

Supplemental Table 1

## Figures and Tables

**Table 1:** Overview of *KDM2B* variants in the cohort

Individual	Variant (NM_032590.4)		Inheritance	gnomAD	In Silico Prediction	EpiSign
Pathogenic variants						
1	c.1912G>A	p.(Gly638Ser)	De novo	−	4/4	Y
3.1, 3.2	c.3370C>T	p.(Arg1124*)	Paternal	−	LoF	Y; Y
4.1, 4.2, 4.3	c.946G>A	p.(Val316Ile)	Paternal	1×	4/4	Y; U; Y
6	c.499C>T	p.(Arg167Trp)	De novo	−	4/4	Y
7	c.1894G>T	p.(Asp632Tyr)	De novo	−	4/4	Y
8	c.1880G>A	p.(Cys627Tyr)	De novo	−	4/4	NA
10	c.457delA	p.(Met153Cysfs*24)	De novo	−	LoF	Y
11	c.3005_3023del19	p.(Asn1002Serfs*35)	De novo	−	LoF	Y
18, 31	c.1847G>A	p.(Cys616Tyr)	De novo	−	4/4	Y; NA
20	c.1913G>A	p.(Gly638Asp)	De novo	−	4/4	NA
22	c.1846T>C	p.(Cys616Arg)	De novo	−	4/4	Y
23	c.1889G>C	p.(Cys630Ser)	De novo	−	4/4	Y
25.1, 25.2	12q24.31 deletion		Paternal	NA	LoF	Y; NA
29	12q24.31 deletion		De novo	NA	LoF	Y
30	12q24.31 deletion		De novo	NA	LoF	Y
Likely pathogenic variants						
13	c.1903_1905delAAG	p.(K635del)	De novo	−	NA	NA
Variants of uncertain significance						
17	c.1627G>A	p.(Ala543Thr)	De novo	2×	4/4	NA
2	c.2297G>A	p.(Arg766Gln)	De novo	2×, 7alt	3/4	N
5.1, 5.2	c.1954A>G	p.(Ile652Val)	Maternal	2×	3/4	N; U
14	c.3637C>T	p.(Arg1213Trp)	De novo	5alt	4/4	N
19	c.777+5G>A	Splice site	De novo	−	3/3 reduced	N

International collaborations resulted in a cohort of 27 individuals representing 21 variants in *KDM2B*. Table 1 indicates genetic details of each variant, appearance of the variant in the gnomAD (or alternative variants affecting the same residu), summary of in silico prediction results, and inclusion in the KDM2B episignature cohort. Additional and supporting information per variant can be found in Supplemental Table 1. “×” indicates times, “−” indicates absence.

*alt*, alternative; *gnomAD*, Genome Aggregation Database; *LoF*, loss-of-function; *N*, no/negative; *NA*, not applicable/not assessed; *U*, uncertain; *Y*, yes/positive.

**Table 2:** An overview of the phenotypes associated with *KDM2B* variants

No.	Sex, Age, y	Variant (NM_032590.4)	Inheritance	ID/DD	Behavior/Psychiatry	Hypotonia	Microcephaly (OFC <−2 SD)	Cardiac Anomalies	Kidney Anomalies	Other
Pathogenic variants										
1	M, 7	c.1912G>A, p.(Gly638Ser)	De novo	Speechdelay, SON-IQ 86	Autism	−	−	VSD, ASD, fetal atrial flutter	−	Familial polydactyly
3.1	F, 7	c.3370C>T, p.(Arg1124*)	Paternal	Severe ID	Hyperactivity	+	−	VSD, DORV	NA	Phelan-McDermid syndrome, 22q13 deletion
3.2	M, 42	c.3370C>T, p.(Arg1124*)	Unknown	Learning difficulties	ADD	NA	−	^−^	NA	COPD
4.1	M, 15	c.946G>A, p.(Val316Ile)	Paternal	Mild	Autism, ADHD, tantrums	NA	−	NA	NA	Epilepsy
4.2	M, 65	c.946G>A, p.(Val316Ile)	Unknown	Learning difficulties, mild ID	NA	NA	−	NA	NA	Decreased renal function, osteoporosis (adult age)
4.3	F, 21	c.946G>A, p.(Val316Ile)	Paternal	Moderate	Autism, tantrums, anxiety	NA	−	NA	NA	
6	M, 9	c.499C>T, p.(Arg167Trp)	De novo	Speech delay, nonverbal IQ 97	ADHD	−	−	−	−	Congenital ptosis, cryptorchidism
7	M, 4	c.1894G>T, p.(Asp632Tyr)	De novo	Learning difficulties	Autism, ADHD, impulsiveness	−	−	PVS, ASD	−	Hypertonia, progressive contractures, inguinal hernia
8	F, 6	c.1880G>A, p.(Cys627Tyr)	De novo	Mild speech delay	−	−	−	ASD, MR, PDA, PVS	Single kidney	Short stature
10	M, 10	c.457del, p.Met153Cysfs*24	De novo	Mild ID, IQ 66	−	−	−	Atrial septal aneurysm, MR	^−^	SHOC2-related Noonan syndrome
11	F, 5	c.3005_3023del19, p.(Asn1002Sfs35)	De novo	Global DD, moderate ID	Autism, hyperactivity	+	−	−	NA	Epilepsy, MRI abnormalities (MCD)
18	M, 5	c.1847G>A, p.(Cys616Tyr)	De novo	Moderate global DD	−	−	+	−	Single kidney	Coloboma, hypertrichosis, failure to thrive
20	F, 14	c.1913G>A, p.(Gly638Asp)	De novo	Speech delay, learning difficulties	Mild autistic features	NA	−	Mild mitral insufficiency	−	Short stature, R oculomotor defect enophtalmus
22	M, 16 mo	c.1846T>C, p.(Cys616Arg)	De novo	Global DD, speech delay	−	Upper limbs	−	PFO	Single kidney	Brain MRI abnormalities, unilateral anophthalmia, bilateral SNHL, facial asymmetry
23	F, 3	c.1889G>C, p.(Cys630Ser)	De novo	Severe DD, no speech	−	+	NA	ASD	Single kidney, right VUR	Short stature, poor weight gain, squint, congenital obstructio ductus nasolacrimalis
25.1	F, 12	12q24.31 deletion (including *KDM2B* and *HNF1A)*	Paternal	Severe, no speech, cannot walk	Not specified	+	+	NA	Normal renal function	Epilepsy, hip dysplasia (Choueryet al,^19^ Krzyzewska et al^20^)
25.2	M, adult	12q24.31 deletion (including *KDM2B* and *HNF1A)*	Unknown	Normal	−	−	−	−	−	Insulin-dependent diabetes at 14 y (Chouery et al,^19^ Krzyzewska et al^20^)
29	F, 12	12q24.31deletion (including *KDM2B* and *SETD1B)*	De novo	+	Autism, ADHD	−	−	NA	NA	Preauricular tags, oligodontia, umbilical hernia (Krzyzewska et al^20^)
30	M, 9	12q24.31 deletion (including *KDM2B* and *SETD1B*)	De novo	+	Probable autism	+	OFC at fourth percentile	NA	NA	Epilepsy (Krzyzewskaet al,^20^ patient 10; Labonne et al^21^)
31	M, 5	c.1847G>A, p.(Cys616Tyr)	De novo	Speech delay, mild-moderate ID	Stereotypies	−	+	ASD	−	Cryptorchidism, talus pes, kyphosis, congenital obstruction of ductus nasolacrimalis
Likely pathogenic variant (sample not available for methylation analysis)										
13	F, 4	c.1903_1905delAAG, p.(Lys635del)	De novo	Moderate speech delay, mild ID	−	+	+	ASD (2 times), PVS, PDA, PFO	−	Feeding difficulties at birth
VUS (sample not available for methylation analysis)										
17	F, 7	c.1627G>A, p.(Ala543Thr)	De novo	+	Autism	+	−	PFO	NA	History of failure to thrive until age 2 y, epilepsy, later obesity, MRI abnormalities
VUS(variants not showing KDM2B specific episignature)										
2	F, 1.8	c.2297G>A, p.(Arg766Gln)	De novo	−	−	−	NA	−	NA	CL/P, preaxial polydactyly, finger contractures, thumb hypoplasia
5.1	M, 28	c.1954A>G, p.(Ile652Val)	Maternal	Mild-moderate	Autism	NA	−	NA	NA	Scoliosis, hearing loss due to cholesteatoma
5.2	F, 58	c.1954A>G, p.(Ile652Val)	Unknown	−	NA	NA	NA	NA	NA	Dyslexia
14	M, 12	c.3637C>T, p.(Arg1213Trp)	De novo	Global DD, limited speech, mild ID (IQ 64)	Hyperactivity, aggressive behavior	+	−	−	−	Macrocephaly, epilepsy, brain MRI abnormalities, hand/finger abnormalities
19	F, 1	c.777+5G>A	De novo	Severe DD, no speech	NA	+	+	ASD	Thrombotic angiopathy	Neonatal seizures, SNHL, abnormal renal vasculature

Table 2 summarizes the clinical features of individuals with *KDM2B* variants. More extensive data are presented in Supplemental Table 2 and the clinical summaries. “+” indicates presence of feature and “−” indicates absence of feature.

*ADD*, attention deficit disorder; *ADHD*, attention deficit hyperactivity disorder; *ASD*, atrial septal defect; *CL/P*, cleft lip/palate; *COPD*, chronic obstructive pulmonary disease; *DD*, developmental delay; *DORV*, double outlet right ventricle; *F*, female; *ID*, intellectual disability; *M*, male; *MCD*, malformation of cortical development; *MR*, mitral regurgitation; *MRI*, magnetic resonance imaging; *NA*, not assessed; *PDA*, persistent ductus arteriosus; *PFO*, persistent foramen ovale; *PVS*, pulmonary valve stenosis; *R*, right; *SNHL*, sensorineural hearing loss; *SON-IQ*, Snijders-Oomen non-verbal intelligence quotient; *VSD*, ventricular septal defect; *VUS*, variant of uncertain significance; *VUR*, vesicoureteral reflux.

## Data Availability

The data that support the findings of this study are available on request from the corresponding author. The data are not publicly available owing to privacy or ethical restrictions.

## References

[1] Fahrner JA, Bjornsson HT. Mendelian disorders of the epigenetic machinery: postnatal malleability and therapeutic prospects. Hum Mol Genet. 2019;28(R2):R254–R264. Published correction appears in Hum Mol Genet. 2020;29(5):876. 10.1093/hmg/ddz174 31595951 PMC6872430

[2] Aref-Eshghi E, Kerkhof J, Pedro VP, Evaluation of DNA methylation episignatures for diagnosis and phenotype correlations in 42 Mendelian neurodevelopmental disorders. Am J Hum Genet. 2020;106(3):356–370. Published correction appearance in Am J Hum Genet. 2021;108(6):1161–1163. 10.1016/j.ajhg.2020.01.019 32109418 PMC7058829

[3] Sadikovic B, Levy MA, Kerkhof J, Clinical epigenomics: genome-wide DNA methylation analysis for the diagnosis of Mendelian disorders. Genet Med. 2021;23(6):1065–1074. Published correction appears in Genet Med. 2021;23(11):2228. 10.1038/s41436-020-01096-4 33547396 PMC8187150

[4] Tsukada Y, Fang J, Erdjument-Bromage H, Histone demethylation by a family of JmjC domain-containing proteins. Nature. 2006;439(7078):811–816. 10.1038/nature04433 16362057

[5] He J, Nguyen AT, Zhang Y. KDM2b/JHDM1b, an H3K36me2-specific demethylase, is required for initiation and maintenance of acute myeloid leukemia. Blood. 2011;117(14):3869–3880. 10.1182/blood-2010-10-312736 21310926 PMC3083299

[6] Kang JY, Kim JY, Kim KB, KDM2B is a histone H3K79 demethylase and induces transcriptional repression via sirtuin-1-mediated chromatin silencing. FASEB J. 2018;32(10):5737–5750. 10.1096/fj.201800242R 29763382

[7] Frescas D, Guardavaccaro D, Bassermann F, Koyama-Nasu R, Pagano M. JHDM1B/FBXL10 is a nucleolar protein that represses transcription of ribosomal RNA genes. Nature. 2007;450(7167):309–313. 10.1038/nature06255 17994099

[8] Janzer A, Stamm K, Becker A, Zimmer A, Buettner R, Kirfel J. The H3K4me3 histone demethylase Fbxl10 is a regulator of chemokine expression, cellular morphology, and the metabolome of fibroblasts. J Biol Chem. 2012;287(37):30984–30992. 10.1074/jbc.M112.341040 22825849 PMC3438931

[9] Klose RJ, Kallin EM, Zhang Y. JmjC-domain-containing proteins and histone demethylation. Nat Rev Genet. 2006;7(9):715–727. 10.1038/nrg1945 16983801

[10] He J, Shen L, Wan M, Taranova O, Wu H, Zhang Y. Kdm2b maintains murine embryonic stem cell status by recruiting PRC1 complex to CpG islands of developmental genes. Nat Cell Biol. 2013;15(4):373–384. 10.1038/ncb2702 23502314 PMC4078788

[11] He J, Kallin EM, Tsukada YI, Zhang Y. The H3K36 demethylase Jhdm1b/Kdm2b regulates cell proliferation and senescence through p15(Ink4b). Nat Struct Mol Biol. 2008;15(11):1169–1175. 10.1038/nsmb.1499 18836456 PMC2612995

[12] Boulard M, Edwards JR, Bestor TH. FBXL10 protects polycomb-bound genes from hypermethylation. Nat Genet. 2015;47(5):479–485. 10.1038/ng.3272 25848754

[13] Deiktakis EE, Abrams M, Tsapara A, Identification of structural elements of the lysine specific demethylase 2B CxxC domain associated with replicative senescence bypass in primary mouse cells. Protein J. 2020;39(3):232–239. 10.1007/s10930-020-09895-z 32270414

[14] Xu C, Liu K, Lei M, DNA sequence recognition of human CXXC domains and their structural determinants. Structure. 2018;26(1):85–95e3. 10.1016/j.str.2017.11.022 29276034

[15] Farcas AM, Blackledge NP, Sudbery I, KDM2B links the polycomb repressive complex 1 (PRC1) to recognition of CpG islands. eLife. 2012;1:e00205. 10.7554/eLife.00205 23256043 PMC3524939

[16] Wu X, Johansen JV, Helin K. Fbxl10/Kdm2b recruits polycomb repressive complex 1 to CpG islands and regulates H2A ubiquitylation. Mol Cell. 2013;49(6):1134–1146. 10.1016/j.molcel.2013.01.016 23395003

[17] Jiang Y, Qian X, Shen J, Local generation of fumarate promotes DNA repair through inhibition of histone H3 demethylation. Nat Cell Biol. 2015;17(9):1158–1168. Published correction appears in Nat Cell Biol. 2018;20(10):1226. 10.1038/ncb3209 26237645 PMC4800990

[18] Marcon E, Ni Z, Pu S, Human-chromatin-related protein interactions identify a demethylase complex required for chromosome segregation. Cell Rep. 2014;8(1):297–310. 10.1016/j.celrep.2014.05.050 24981860

[19] Chouery E, Choucair N, Abou Ghoch J, El Sabbagh S, Corbani S, Mégarbané A. Report on a patient with a 12q24.31 microdeletion inherited from an insulin-dependent diabetes mellitus father. Mol Syndromol. 2013;4(3):136–142. 10.1159/000346473 23653585 PMC3638924

[20] Krzyzewska IM, Maas SM, Henneman P, A genome-wide DNA methylation signature for SETD1B-related syndrome. Clin Epigenetics. 2019;11(1):156. 10.1186/s13148-019-0749-3 31685013 PMC6830011

[21] Labonne JDJ, Lee KH, Iwase S, An atypical 12q24.31 microdeletion implicates six genes including a histone demethylase KDM2B and a histone methyltransferase SETD1B in syndromic intellectual disability. Hum Genet. 2016;135(7):757–771. 10.1007/s00439-016-1668-4 27106595

[22] Girard SL, Gauthier J, Noreau A, Increased exonic de novo mutation rate in individuals with schizophrenia. Nat Genet. 2011;43(9):860–863. 10.1038/ng.886 21743468

[23] Monies D, Abouelhoda M, AlSayed M, The landscape of genetic diseases in Saudi Arabia based on the first 1000 diagnostic panels and exomes. Hum Genet. 2017;136(8):921–939. 10.1007/s00439-017-1821-8 28600779 PMC5502059

[24] Yokotsuka-Ishida S, Nakamura M, Tomiyasu Y, Positional cloning and comprehensive mutation analysis identified a novel KDM2B mutation in a Japanese family with minor malformations, intellectual disability, and schizophrenia. J Hum Genet. 2021;66(6):597–606. 10.1038/s10038-020-00889-4 33402700

[25] Sobreira N, Schiettecatte F, Valle D, Hamosh A. GeneMatcher: a matching tool for connecting investigators with an interest in the same gene. Hum Mutat. 2015;36(10):928–930. 10.1002/humu.22844 26220891 PMC4833888

[26] Schwarz JM, Cooper DN, Schuelke M, Seelow D. MutationTaster2: mutation prediction for the deep-sequencing age. Nat Methods. 2014;11(4):361–362. 10.1038/nmeth.2890 24681721

[27] Wiel L, Baakman C, Gilissen D, Veltman JA, Vriend G, Gilissen C. MetaDome: pathogenicity analysis of genetic variants through aggregation of homologous human protein domains. Hum Mutat. 2019;40(8):1030–1038. 10.1002/humu.23798 31116477 PMC6772141

[28] Adzhubei IA, Schmidt S, Peshkin L, A method and server for predicting damaging missense mutations. Nat Methods. 2010;7(4):248–249. 10.1038/nmeth0410-248 20354512 PMC2855889

[29] Vaser R, Adusumalli S, Leng SN, Sikic M, Ng PC. SIFT missense predictions for genomes. Nat Protoc. 2016;11(1):1–9. 10.1038/nprot.2015.123 26633127

[30] Richards S, Aziz N, Bale S, Standards and guidelines for the interpretation of sequence variants: a joint consensus recommendation of the American College of Medical Genetics and Genomics and the Association for Molecular Pathology. Genet Med. 2015;17(5):405–424. 10.1038/gim.2015.30 25741868 PMC4544753

[31] Karczewski KJ, Francioli LC, Tiao G, The mutational constraint spectrum quantified from variation in 141,456 humans. Nature. 2020;581(7809):434–443. Published correction appears in Nature. 2021;590(7846):E53. Published correction appears in Nature. 2021;597(7874):E3-E4. 10.1038/s41586-020-2308-7 32461654 PMC7334197

[32] Andricovich J, Kai Y, Peng W, Foudi A, Tzatsos A. Histone demethylase KDM2B regulates lineage commitment in normal and malignant hematopoiesis. J Clin Invest. 2016;126(3):905–920. 10.1172/JCI84014 26808549 PMC4767361

[33] Blackledge NP, Farcas AM, Kondo T, Variant PRC1 complex-dependent H2A ubiquitylation drives PRC2 recruitment and polycomb domain formation. Cell. 2014;157(6):1445–1459. 10.1016/j.cell.2014.05.004 24856970 PMC4048464

[34] Fukuda T, Tokunaga A, Sakamoto R, Yoshida N. Fbxl10/Kdm2b deficiency accelerates neural progenitor cell death and leads to exencephaly. Mol Cell Neurosci. 2011;46(3):614–624. 10.1016/j.mcn.2011.01.001 21220025

[35] Testoni S, Bartolone E, Rossi M, KDM2B is implicated in bovine lethal multi-organic developmental dysplasia. PLoS One. 2012;7(9):e45634. 10.1371/journal.pone.0045634 23029151 PMC3459949

[36] Aref-Eshghi E, Bend EG, Colaiacovo S, Diagnostic utility of genome-wide DNA methylation testing in genetically unsolved individuals with suspected hereditary conditions. Am J Hum Genet. 2019;104(4):685–700. 10.1016/j.ajhg.2019.03.008 30929737 PMC6451739

[37] Aref-Eshghi E, Rodenhiser DI, Schenkel LC, Genomic DNA methylation signatures enable concurrent diagnosis and clinical genetic variant classification in neurodevelopmental syndromes. Am J Hum Genet. 2018;102(1):156–174. 10.1016/j.ajhg.2017.12.008 29304373 PMC5777983

[38] Qiao Y, Tyson C, Hrynchak M, Clinical application of 2.7M Cytogenetics array for CNV detection in subjects with idiopathic autism and/or intellectual disability. Clin Genet. 2013;83(2):145–154. 10.1111/j.1399-0004.2012.01860.x 22369279

[39] Boulard M, Edwards JR, Bestor TH. Abnormal X chromosome inactivation and sex-specific gene dysregulation after ablation of FBXL10. Epigenetics Chromatin. 2016;9:22. 10.1186/s13072-016-0069-1 27252784 PMC4888662

[40] Winkelmann J, Lin L, Schormair B, Mutations in DNMT1 cause autosomal dominant cerebellar ataxia, deafness and narcolepsy. Hum Mol Genet. 2012;21(10):2205–2210. 10.1093/hmg/dds035 22328086 PMC3465691

[41] Sun L, Huang L, Nguyen P, DNA methyltransferase 1 and 3B activate BAG-1 expression via recruitment of CTCFL/BORIS and modulation of promoter histone methylation. Cancer Res. 2008;68(8):2726–2735. 10.1158/0008-5472.CAN-07-6654 18413740 PMC2733164

[42] Hunter AGW, Dupont B, McLaughlin M, The Hunter-McAlpine syndrome results from duplication 5q35-qter. Clin Genet. 2005;67(1):53–60. 10.1111/j.1399-0004.2005.00378.x 15617549

[43] Hunter AG, McAlpine PJ, Rudd NL, Fraser FC. A “new” syndrome of mental retardation with characteristic facies and brachyphalangy. J Med Genet. 1977;14(6):430–437. 10.1136/jmg.14.6.430 342696 PMC1013640

[44] Rayasam GV, Wendling O, Angrand PO, NSD1 is essential for early post-implantation development and has a catalytically active SET domain. EMBO J. 2003;22(12):3153–3163. 10.1093/emboj/cdg288 12805229 PMC162140

[45] Zech M, Boesch S, Maier EM, Haploinsufficiency of KMT2B, encoding the lysine-specific histone methyltransferase 2B, results in early-onset generalized dystonia. Am J Hum Genet. 2016;99(6):1377–1387. 10.1016/j.ajhg.2016.10.010 27839873 PMC5142117

[46] Demers C, Chaturvedi CP, Ranish JA, Activator-mediated recruitment of the MLL2 methyltransferase complex to the beta-globin locus. Mol Cell. 2007;27(4):573–584. 10.1016/j.molcel.2007.06.022 17707229 PMC2034342

[47] Ciolfi A, Foroutan A, Capuano A, Childhood-onset dystonia-causing KMT2B variants result in a distinctive genomic hypermethylation profile. Clin Epigenetics. 2021;13(1):157. 10.1186/s13148-021-01145-y 34380541 PMC8359374

[48] Kernohan KD, Cigana Schenkel L, Huang L, Identification of a methylation profile for DNMT1-associated autosomal dominant cerebellar ataxia, deafness, and narcolepsy. Clin Epigenetics. 2016;8:91. 10.1186/s13148-016-0254-x 27602171 PMC5011850

[49] Gao Y, Duque-Wilckens N, Aljazi MB, Impaired KDM2B-mediated PRC1 recruitment to chromatin causes defective neural stem cell self-renewal and ASD/ID-like behaviors. iScience. 2022;25(2):103742. 10.1016/j.isci.2022.103742 35128353 PMC8800019

